# A Quantitative Comparison of Medial and Coronal Dentate Gyrus Microdissection Strategies and a Softening-Based Workflow for Reproducible Tissue Procurement

**DOI:** 10.3390/life16030511

**Published:** 2026-03-20

**Authors:** Turan Koç, Nail Can Öztürk

**Affiliations:** 1 Department of Anatomy, Faculty of Medicine, Kahramanmaraş Sütçü İmam University, Kahramanmaraş 46050, Turkey; 2 Department of Anatomy, Faculty of Medicine, Mersin University, Mersin 33343, Turkey

**Keywords:** dentate gyrus, microdissection, neuroanatomical methods, tissue softening, reproducibility, adult hippocampal neurogenesis

## Abstract

A reliable isolation of the dentate gyrus (DG) is a critical pre-analytical step for region-specific neurobiological assays, yet DG microdissection practices vary widely and are rarely compared quantitatively under standardized conditions. In addition, long-term paraformaldehyde-fixed archival brain tissue is commonly regarded as unsuitable for microdissection because of reduced pliability and poor anatomical contrast, limiting its use for training and protocol development. Here, we quantitatively compare two commonly used DG microdissection strategies, a medial (intact-block) approach and a coronal (slice-guided) approach across fresh, fixed, and softened-fixed rat brain hemispheres under matched conditions. To enable the use of archival material, fixed hemispheres were subjected to a simple 15-day slow-running tap water softening protocol to improve tissue handling and landmark visibility. Dissection duration and anatomical specificity were evaluated, the latter quantified by measuring residual cornu ammonis (CA)1–3 area on hematoxylin–eosin-stained coronal sections following DG removal. In fresh tissue, the medial approach enabled significantly faster DG isolation than the coronal approach, while both strategies achieved comparable anatomical specificity. In softened-fixed tissue, dissection times increased for both approaches, but the same relative performance ranking was preserved. Softening markedly improved tissue pliability and boundary visualization, particularly benefiting the coronal, stepwise dissection strategy. Residual CA1–3 areas did not differ significantly between approaches or tissue states. This study provides a validated, training-oriented DG microdissection workflow that supports methodological standardization, reproducibility, and 3R-aligned use of archival tissue, strengthening the pre-analytical foundation for downstream region-specific neuroscience assays.

## 1. Introduction

Reliable isolation of anatomically defined brain regions is a critical pre-analytical step for a wide range of neuroscience applications, including region-specific transcriptomic, proteomic, and histological analyses. Variability introduced during tissue procurement can substantially influence downstream data quality, reproducibility, and cross-study comparability. Despite this, microdissection procedures are often underreported or insufficiently standardized, even when targeting small and anatomically complex brain structures. Defining precise anatomical boundaries can be challenging in complex neurobiological systems, particularly where neural, immune, and vascular components interact closely. This broader context highlights the importance of carefully designed anatomical workflows, such as the DG microdissection protocol described in the present study.

The dentate gyrus (DG), a sub-region of the hippocampus (HP), presents challenges for reproducible isolation owing to its curved geometry, close proximity to cornu ammonis 1–3 (CA1–CA3) subfields, and pronounced dorsal–ventral heterogeneity [1,2]. Accurate delineation of DG boundaries is therefore essential to minimize unintended inclusion of adjacent hippocampal regions, which may bias region-specific molecular and anatomical analyses. Recent spatial transcriptomic studies of the human DG have shown that its sub-regions, including the granule cell layer (GCL), sub-granular zone (SGZ), and CA4/hilus, display distinct spatial and transcriptional profiles. This spatial heterogeneity highlights the importance of careful anatomical sampling when investigating DG sub-regions [3]. Although immunohistochemical approaches on sectioned brain tissue remain widely used to study DG organization and cellular composition [4,5], downstream molecular investigations increasingly require freshly isolated DG to preserve nucleic acid and protein integrity [6].

Adult hippocampal neurogenesis (AHN), which occurs within the DG sub-granular zone, represents one of several research contexts in which precise DG procurement is particularly important [7,8,9,10]. AHN contributes to hippocampal plasticity and has been implicated in cognitive processes and neurological disease [11,12]. However, irrespective of the downstream biological question, reproducible DG isolation remains a shared methodological prerequisite across diverse experimental paradigms.

Only a limited number of studies provide detailed methodological guidance for DG microdissection. Among these, Hagihara et al. [6] described a medial, intact-block DG dissection protocol optimized for fresh tissue. In contrast, DG isolation described in other studies has typically occurred as part of broader experimental workflows rather than as explicitly validated dissection methods [13,14]. As a result, coronal slice-based approaches are widely used but have not been formally standardized or quantitatively compared with medial dissection strategies under matched anatomical conditions.

An additional practical limitation concerns the use of long-term paraformaldehyde-fixed archival brain tissue for DG microdissection. Fixed tissue is often considered suboptimal due to increased rigidity, fragmentation, and loss of natural color contrast required for reliable identification of hippocampal landmarks. This restricts its use for training and method development, thereby increasing reliance on freshly sacrificed animals. Simple approaches that restore tissue pliability and landmark visibility in archival specimens could therefore provide methodological and ethical advantages.

In the present study, we performed a controlled, side-by-side comparison of medial and coronal DG microdissection strategies across fresh, fixed, and softened-fixed rat brain hemispheres. We employed a prolonged slow-running tap water softening protocol to improve handling properties and anatomical visibility in archival fixed tissue, explicitly for training and anatomical validation purposes. Dissection performance was evaluated using two method-relevant outcome measures: (i) time required for DG isolation and (ii) anatomical specificity, quantified by residual CA1–CA3 area on histological sections following DG removal. By focusing on anatomical feasibility, procedural performance, and reproducibility rather than molecular endpoints, this work provides a validated framework for DG microdissection that supports methodological standardization, training, and Reduction and Refinement principles within the 3Rs.

## 2. Materials and Methods

### 2.1. Animals

We used 21 adult female Sprague Dawley (RRID: RRRC_00239) rats (6–7 months; ~190–210 g). All animals were housed under standard laboratory conditions. Animals were kept in polypropylene cages containing wood-chip bedding in a controlled environment (22 ± 2 °C, 50–60% relative humidity). The animals were maintained on a 12 h light/dark cycle with lights on at 07:00 a.m. Standard laboratory chow and tap water were available *ad libitum* throughout the study. For fixed cohorts, rats were perfused with saline followed by ice-cold 4% PFA in 0.1 M PBS. Brains were immersion-fixed for 48 h in 4% PFA, then stored for four years at 4 °C in 0.1–0.5% PFA in PBS. Fresh hemispheres were obtained under deep ketamine/xylazine anesthesia.

### 2.2. Experimental Design

Seven groups were defined (n = 3 hemispheres per group):

(1) Control (no DG removal) a positive control group in the histological assessment of DG dissection success; (2) fixed medial (FXM), PFA-fixed hemispheres, dissected with the medial approach; (3) fixed coronal (FXC), PFA-fixed hemispheres, dissected with the coronal approach; (4) softened medial (SM), PFA-fixed hemispheres that were subjected to softening procedure, dissected with the medial approach; (5) softened coronal (SC), PFA-fixed hemispheres that were subjected to softening procedure, dissected with the coronal approach; (6) fresh medial (FM), freshly held hemispheres, dissected with the medial approach; (7) fresh coronal (FC), freshly held hemispheres, dissected with the coronal approach (Table 1). However, the fixed groups [fixed medial (FXM) and fixed coronal (FXC), n = 6] were not included in the quantitative comparisons of dissection duration or excised region area because the pronounced tissue rigidity and reduced visibility of anatomical boundaries in fixed specimens prevented reliable evaluation of the microdissection procedure. Consequently, the final quantitative analysis was performed in five experimental groups with three hemispheres per group (n = 3). This sample size was selected in the context of an exploratory methodological/anatomical study and is in line with commonly used principles in animal experimentation, including the Mead resource equation approach, which supports a minimum of three animals per group in exploratory designs.

### 2.3. Isolation of Brain Hemispheres

**Figure 1:**
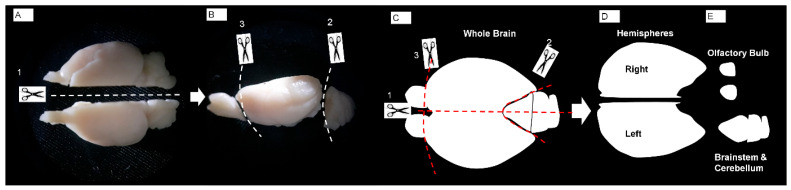
Stepwise procedures for obtaining brain hemispheres are summarized in panels (**A**–**E**). Incision sites are indicated by scissor icons and labeled as steps 1–3.

Brain hemispheres were isolated using a standardized dissection procedure. First, a midline incision was made along the longitudinal fissure to divide the whole brain into two hemispheres (Figure 1A,C,D). The brainstem and cerebellum were then carefully separated from the forebrain to obtain the cerebral hemispheres (Figure 1B,C,E). Finally, the olfactory bulbs were transected at the coronal plane, completing the hemispheric isolation (Figure 1B,C,E).

### 2.4. Brain Hemisphere Softening Procedure

Archival PFA-fixed hemispheres were softened by immersion in slow-running tap water at room temperature for 15 days prior to DG microdissection. This procedure was adapted from the slow-running water method described by Suzuki et al. [15], originally applied for a one-week period to reduce fixative excess in histopathology contexts. In preliminary trials in our laboratory, one week of rinsing did not sufficiently improve tissue pliability or restore hippocampal color contrast for consistent DG dissection; therefore, the duration was empirically extended to 15 days.

The water flow was checked periodically to ensure continuous rinsing throughout the procedure. No chemical preservatives were added, as the softened hemispheres were intended solely for anatomical training and pilot histological evaluation rather than molecular or genetic analyses. This simple, low-cost approach provides improved pliability and landmark visibility in long-term-fixed tissue and allows trainees to practice DG isolation using archival material commonly available in neuroscience laboratories, without requiring additional animals or specialized equipment.

### 2.5. DG Dissection via Medial Approach

**Figure 2:**
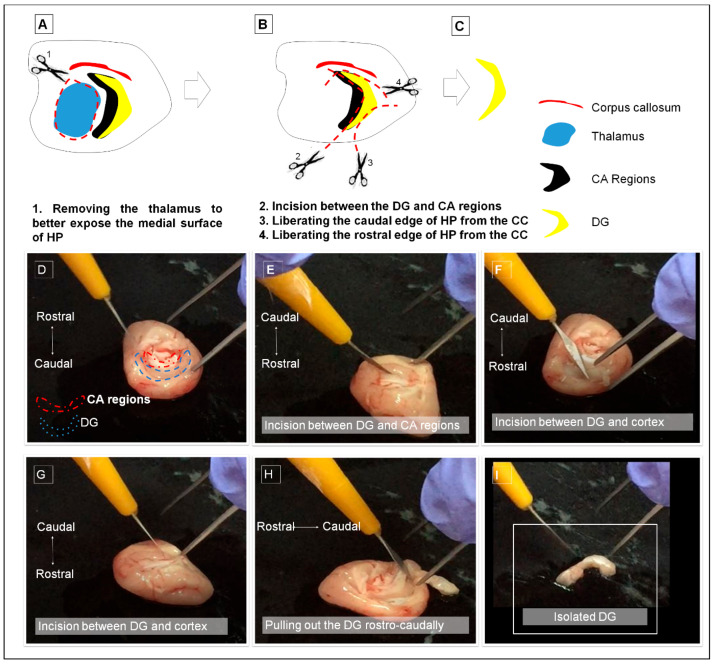
. Stepwise procedures for isolating the dentate gyrus (DG) using the medial approach are illustrated in panels (**A**–**C**). Sequential images captured from the dissection video are shown in panels (**D**–**I**). The incision points are indicated with scissor icons in panels (**A**,**B**). HP hippocampus, DG dentate gyrus, CA cornu ammonis, CC cerebral cortex.

All microdissection procedures in the present study were performed by a single trained operator under standardized experimental conditions. We adapted the medial side dissection protocol from Hagihara et al. [6] (Supplementary Videos S1–S3, Figure 2).

### 2.6. DG Dissection via Coronal Approach

**Figure 3:**
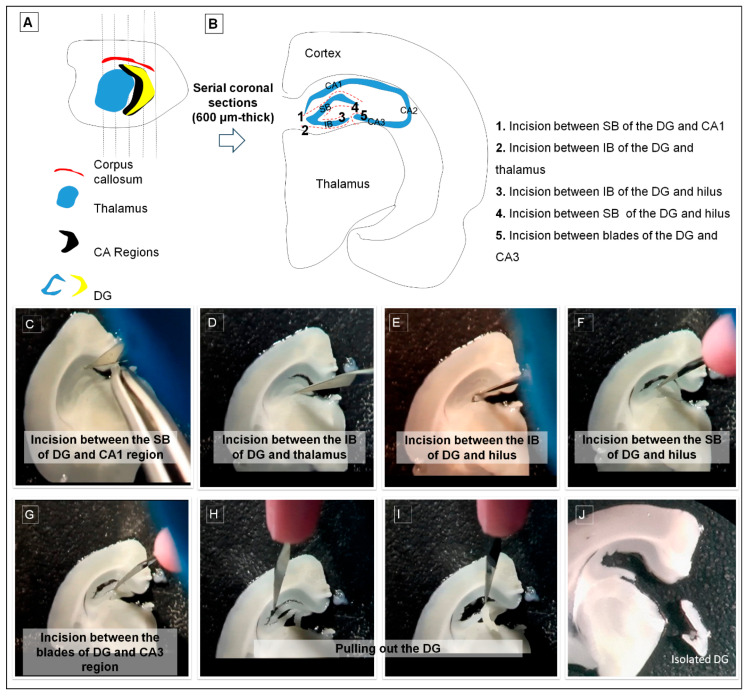
Stepwise procedures for isolating the dentate gyrus (DG) using the coronal approach are illustrated in panels (**A**,**B**). Sequential frames from the dissection video are presented in panels (**C**–**J**). Each operational step is numbered in panel (**B**) and described in the figure. Isolated dentate gyrus (DG) tissue is shown in the rectangle (**J**). DG dentate gyrus, CA cornu ammonis, IB infra blade, SB supra blade.

We reconstructed a coronal slice-based DG dissection strategy based on descriptions in Gilley et al. [13] and Guo et al. [14] (Supplementary Videos S4–S6; Figure 3). Prior to dissection, each brain hemisphere was placed on a stable platform positioned over an ice bucket, and 600 μm thick coronal slices were rapidly sectioned (Figure 3A).

Following sectioning, the dissection proceeded through the following steps, which are illustrated in Figure 3.

### 2.7. Vibratome Sectioning

We performed vibratome sectioning either prior to applying the medial approach or following the coronal dissection, depending on the study group (as summarized in the experimental design; see Table 1). The procedure was adapted from previously published protocols [16,17]. We embedded brain hemispheres in 1% (*w*/*v*) agarose and sectioned into 600 μm thick coronal slices using a vibratome (RRID:SCR_016495) (Leica Microsystems, Buffalo Grove, IL, USA) (see Supplementary Video S7).

### 2.8. Outcome Measures

1. Dissection duration: time required (seconds) to complete DG isolation from one hemisphere.
2. Residual CA area: CA1–CA3 area remaining on hematoxylin and eosin (H&E)-stained coronal sections after DG removal, quantified using the freehand selection tool in ImageJ (2.17.0) (RRID:SCR_003070).

DG area was also measured to provide additional anatomical context.

### 2.9. Hematoxylin and Eosin Staining

H&E staining was performed on 600 µm thick coronal slices from DG-excised hemispheres to assess the CA1–CA3 regions across experimental groups.

### 2.10. Imaging and Video Capturing

Each experimental group contained n = 3 rats, and one 600 µm coronal slice per rat was used for analysis. Residual CA1–CA3 area was quantified on this slice using the freehand selection tool in ImageJ. To document the full H&E-stained coronal slice, several adjacent microscope fields were captured using an Olympus SZ51 light microscope equipped with an Olympus LC30 digital camera (Olympus Optical Company Ltd., Tokyo, Japan), and montaged into a single composite image. Dissection procedure videos were assembled in Microsoft Clipchamp, and playback speed was doubled to facilitate visualization of procedural steps.

### 2.11. Statistical Analysis

Statistical analyses were performed using IBM SPSS Statistics 26 software (RRID:SCR_016479). Normality and homogeneity of variance were assessed for each dataset using the Shapiro–Wilk and Levene’s tests, respectively. As these assumptions were not violated, normally distributed dependent variables were analyzed using the independent-samples *t*-test or one-way ANOVA, as appropriate. Results are presented as mean ± SD, together with the corresponding test statistics, degrees of freedom, and *p*-values. For independent-samples *t*-tests, 95% confidence intervals (CIs) of the mean differences were also reported. In addition, partial eta squared (η^2^p) was reported as an estimate of effect size for both *t*-test- and ANOVA-based comparisons. η^2^p reflects the proportion of variance in the dependent variable attributable to the group effect; however, because effect size estimates may be inflated in small-sample designs, these values were interpreted cautiously. Reporting η^2^p may nevertheless support the interpretation of practical significance and facilitate future power calculations and meta-analytic comparisons. A *p*-value < 0.05 was considered statistically significant.

## 3. Results

### 3.1. Visibility and Handling in Archival Tissues

**Figure 4:**
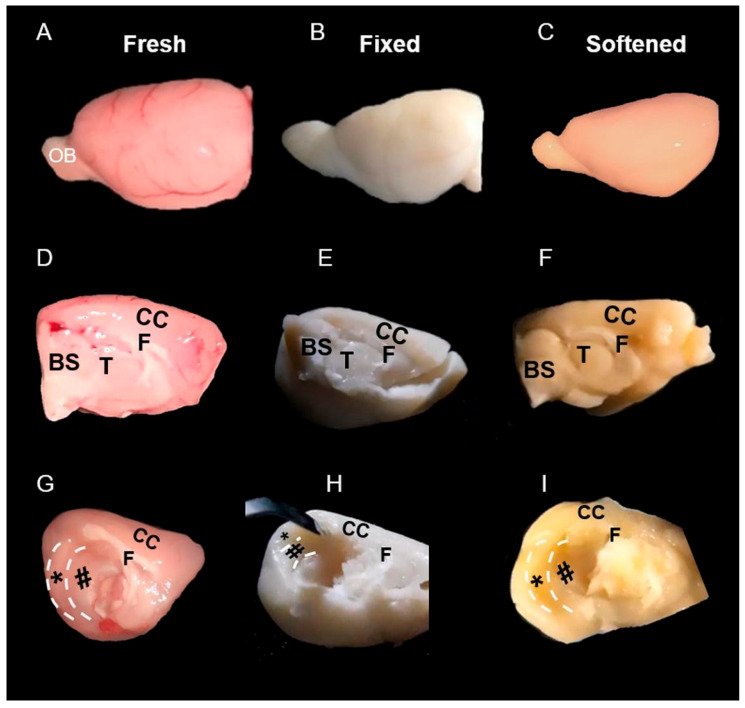
Lateral and medial surfaces of the brain hemispheres from the fresh, fixed, and softened groups are shown in panels (**A**–**C**) and (**D**–**F**), respectively. Panels (**G**–**I**) display the medial surfaces of the hemispheres during dissection using the medial approach. BS brainstem, CC cerebral cortex, F fornix, T thalamus, OB olfactory bulb, (*): Dentate gyrus, (#): Cornu ammonis (CA1–CA3).

**Figure 5:**
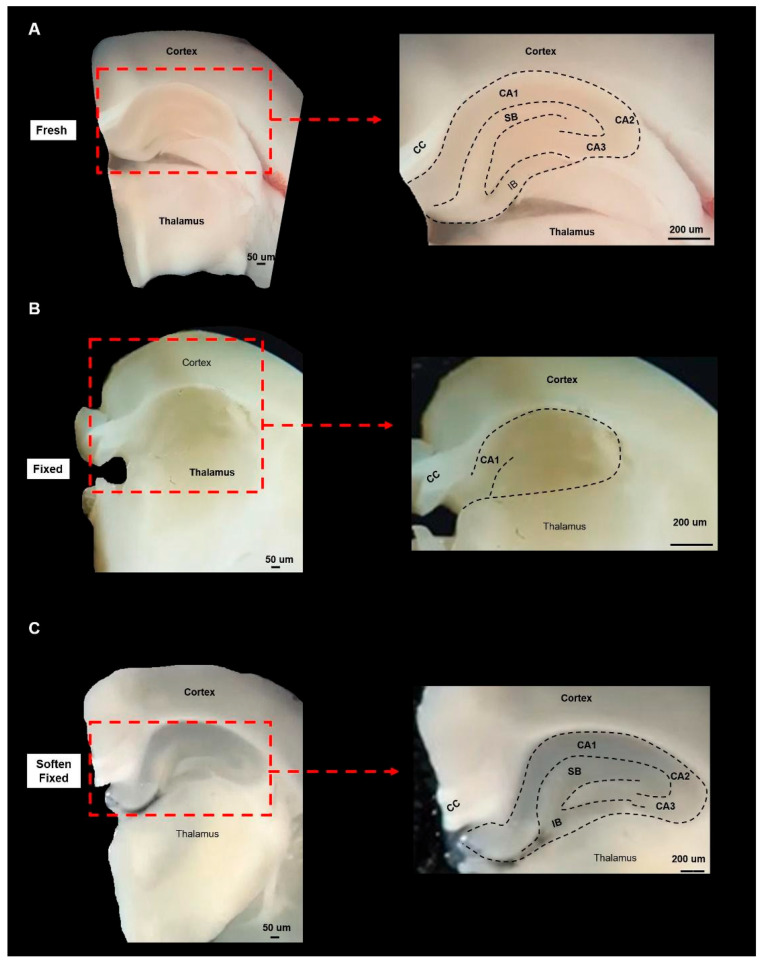
Coronal brain slices obtained from fresh, fixed, and softened brain hemispheres are shown in panels (**A**–**C**). Enlarged views of the hippocampal region, indicated by red rectangular boxes in each panel, are also presented (**A**–**C**) to highlight anatomical distinctions. CA cornu ammonis regions, CC corpus callosum, IB infra blade, SB supra blade.

When we attempted to practice the coronal [13] and medial [6] DG dissection approaches on 4% PFA-fixed brain hemispheres with long-term archival storage, we found that the tissues were markedly rigid and prone to fragmentation, making precise manipulation difficult (Supplementary Videos S2 and S5). Due to pronounced alterations in tissue color and contrast (Figure 4A–C), demarcating the borders of the hippocampus HP and the sub-regions on both the medial surface (Figure 4D,E) and coronal slices (Figure 5B) was substantially more challenging compared with fresh brains.

### 3.2. Softening Restores Visibility and Handling in Archival Tissue

The 15-day softening improved tissue pliability and enhanced the contrast of anatomical landmarks, increasing the visibility of hippocampal borders on both medial surfaces and coronal slices. Ease of manipulation improved, particularly for the coronal approach. On the 15th day of the procedure, we detected marked color change in the HP as compared to the fixed hemispheres (Figure 4B,C). On the medial surface of the softened hemispheres, we saw that the borders of HP and surrounding structures became readily visible as compared to the fixed brain hemispheres (Figure 4E,F). We further observed a marked improved visibility of the neuroanatomical borders in the fixed vs. softened brains in the coronal slices (Figure 5B,C). After the softening procedure, the ease of tissue manipulation improved during coronal dissection (Supplementary Video S6), tissue pliability on the medial surface remained somewhat limited compared to fresh hemispheres (Supplementary Video S3). All measurements underwent normality and variance testing (Shapiro–Wilk and Levene’s tests). Because assumptions were not violated, independent-samples t tests and one-way ANOVA were used for normally distributed variables.

### 3.3. Dissection Duration: Medial Is Faster than Coronal

In the fresh and softened-fixed brains, we recorded the total dissection duration to remove the DG from each hemisphere using both approaches (Table 2 and Table 3). On fresh hemispheres, medial dissection was significantly faster than coronal (FM vs. FC: 51.67 ± 6.51 s vs. 125.33 ± 8.50 s; t(4) = −11.80; *p* = 0.00029). A similar pattern was observed in softened-fixed hemispheres (SM vs. SC: 301.66 ± 12.34 s vs. 727.33 ± 16.62 s; t(4) = −35.61; *p* = 3.71 × 10^−6^). As expected, both approaches were slower in softened-fixed tissue than fresh (FM vs. SM: t(4) = −31.01; *p* = 6.43 × 10^−6^; FC vs. SC: t(4) = −55.68; *p* = 6.22 × 10^−7^).

### 3.4. Anatomical Specificity: Comparable Across Strategies and Tissue States

**Figure 6:**
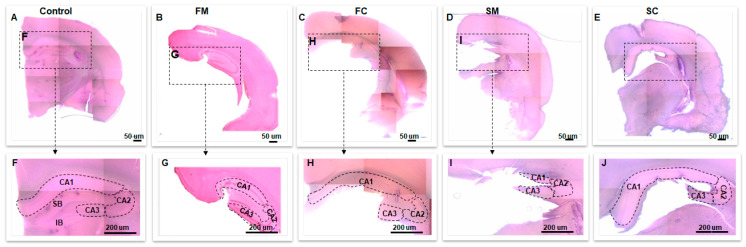
Representative hematoxylin and eosin (H&E)-stained coronal brain slices are shown for each experimental group (**A**–**E**). These images were generated by montaging multiple micrographs acquired using a 4× objective lens. Enlarged views of the boxed regions in panels (**A**–**E**) are presented in panels (**F**–**J**), respectively, to highlight hippocampal sub-regions. CA cornu ammonis regions, FC fresh coronal, FM fresh medial, IF infra-blade, SP supra-blade, SM softened medial, SC softened coronal.

**Figure 7:**
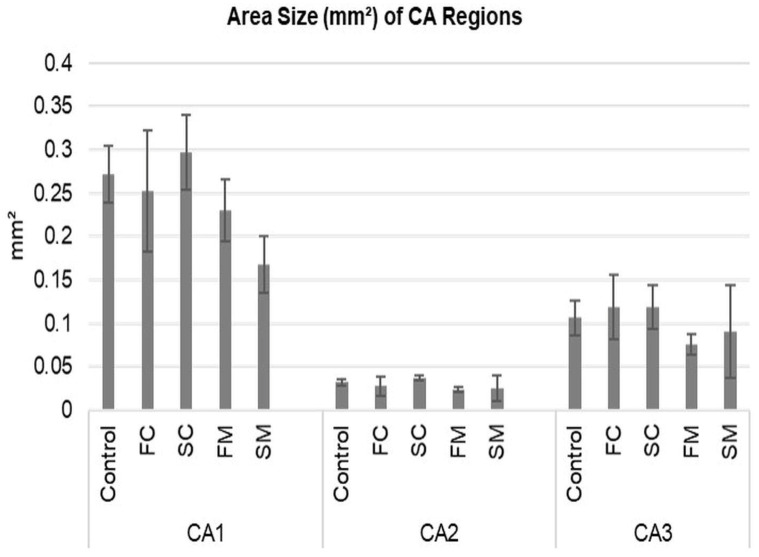
The surface areas (mm^2^) of the cornu ammonis (CA) regions across experimental groups are presented as bar graphs showing mean ± standard deviation (SD). Surface areas refer to the CA region area shown in Figure 6F–J. Statistical analysis using a one-way ANOVA revealed no significant differences among the groups for each CA sub-region. FC fresh coronal, FM fresh medial, SM softened medial, SC softened coronal.

To assess anatomical specificity of DG dissection, we measured CA1–CA3 areas on the H&E-stained 600 μm coronal slices obtained after DG removal. We grouped the measurements as follows:

Control (no DG removal) (Figure 6A), FM (fresh medial) (Figure 6B), FC (fresh coronal) (Figure 6C), SM (softened medial) (Figure 6D), SC (softened coronal) (Figure 6E) Residual CA1–CA3 areas did not differ significantly across groups (one-way ANOVA, *p* > 0.05; Table 4 and Table 5; Figure 6 and Figure 7), indicating comparable anatomical specificity for medial and coronal strategies across fresh and softened-fixed tissue conditions.

## 4. Discussion

This study provides a direct validation of two commonly used DG microdissection strategies, demonstrating that the medial approach is faster while both medial and coronal approaches achieve comparable anatomical specificity as quantified by residual CA1–CA3 area. By isolating dissection strategy as the primary pre-analytical factor, our results address a long-standing gap affecting reproducibility and comparability in AHN-related studies that depend on precisely procured DG tissue.

### 4.1. Medial vs. Coronal: Visibility vs. Speed

In fresh brains, natural color contrast and tissue pliability made both strategies straightforward to execute; nonetheless, the medial approach was consistently faster (~52 ± 6.5 s) than the coronal approach (~125 ± 8.5 s). Both approaches were readily performed under fresh conditions. By contrast, PFA-fixed tissue was rigid, brittle and showed reduced color contrast, obscuring DG-CA borders and impairing border identification regardless of strategy, consistent with the importance of maintaining clear DG-CA boundaries [6]. Archival fixation and long-term storage may alter tissue consistency and histological contrast, which can make the identification of delicate anatomical landmarks more challenging during microdissection. Previous studies have shown that preservation methods used in anatomical specimens can affect the microscopic integrity of central nervous system tissues and influence the visibility of fine structural details in embalmed neural samples [18]. In addition, experimental disease models have demonstrated that systemic biological conditions may lead to subtle morphological alterations across multiple organs, indicating that tissue microarchitecture can vary depending on underlying biological or preservation-related factors [19]. Taken together, these findings suggest that the clarity of dentate gyrus landmarks in archival material may be reduced following prolonged fixation, thereby potentially increasing the technical difficulty of precise DG microdissection, as observed in the present study. To mitigate this, a 15-day slow-running tap water softening improved pliability and border visibility. After softening, coronal dissections benefitted the most, with handling and visibility approaching the fresh tissue experience; the medial approach remained workable but retained somewhat lower pliability than in fresh tissue due to its reliance on medial surface landmarks during diencephalon removal. As expected, both strategies took longer in softened-fixed than in fresh tissue, but the relative ranking in speed was preserved (medial < coronal), while anatomical specificity remained comparable across approaches. Taken together, a medial approach remains advantageous when minimizing duration or procuring the DG in a single tissue block, whereas a coronal approach may offer greater border visibility and stepwise anatomical control, particularly in softened archival tissue.

### 4.2. 3R-Aligned Training and Workflow Standardization

The 15-day softening protocol provides a practical surrogate for DG microdissection, readily adoptable by many neuroscience laboratories to standardize technique and workflow for DG and other hard to dissect neuroanatomical regions. Prolonged exposure of fixed tissue to aqueous environments may induce mild osmotic swelling or partial relaxation of fixation-induced protein cross-links, which can increase tissue pliability while potentially affecting microstructural contrast [20]. Nevertheless, excessive or uncontrolled softening could potentially introduce subtle distortions, and this should be considered when such preparations are used for training purposes. Importantly, no evident macroscopic anatomical distortion was observed during DG dissection in the present study; however, subtle microstructural alterations were not formally quantified. Therefore, softened archival tissue should primarily be considered a practical surrogate for training and methodological familiarization rather than a direct equivalent of fresh tissue. This supports training and protocol refinement without requiring additional animals. In line with the 3Rs framework (Replacement, Reduction, Refinement) [21], this surrogate reduces reliance on newly sacrificed animals for skill acquisition, supports the use of archival material for training, and refines dissection by improving visibility and pliability. Recent developments in experimental neuroscience have increasingly emphasized the importance of combining biological models with digital analytical tools to improve reproducibility and training in complex research settings. At the same time, these approaches contribute to more ethically responsible research practices. Organoid-based experimental systems, for example, are now often integrated with technologies such as organ-on-chip platforms, computational modeling, and digital twin frameworks. These integrated systems make it possible to study complex biological processes in more controlled and reproducible environments while also supporting the principles of the 3Rs by promoting alternative approaches that can reduce the use of experimental animals. In Alzheimer’s disease research, brain organoids derived from patient-specific induced pluripotent stem cells can reproduce several structural and functional characteristics of the human brain. When combined with digital modelling strategies, these systems provide valuable opportunities for investigating disease mechanisms while also contributing to the Replacement and Reduction in animal models in neuroscience research [22]. In a similar way, the integration of biological and digital tools may also support the standardization of DG microdissection training and the optimization of experimental workflows, allowing trainees to develop technical skills in more controlled and ethically sound learning environments.

### 4.3. Implications for AHN Workflows

For AHN studies, precise DG procurement is critical because the DG shows robust dorsal–ventral molecular and epigenetic differentiation [23,24,25], along with cell and circuit level differences including divergent mossy cell projections and synaptic modulation along the axis [26,27]. Variation in dissection boundaries or procedural time can therefore plausibly bias downstream molecular readouts and histological quantification. Our direct comparison indicates that strategy choice affects speed and ease of manipulation without compromising anatomical specificity. Although we did not assess molecular endpoints, choosing a medial approach when minimizing ex vivo duration is important, and selecting a coronal approach when maximizing boundary visibility or targeting specific dorso-ventral levels is needed may help reduce pre-analytical variability in AHN workflows. This recommendation is motivated by robust dorso-ventral differences in DG [23,24,25,26,27].

### 4.4. Considerations Related to Molecular Integrity

Regarding the degradation of nucleic acids and proteins, differences in dissection duration between approaches may be considered if molecular preservation is a priority. The storage temperature and duration are known to have an impact on the RNA integrity [28]. However, Catts and colleagues detected no significant change on RNA yield, purity or 28S/18S optical density at six-hour postmortem in mouse brain tissue [29]. Therefore, the approximately one-minute difference between approaches observed here is unlikely to markedly influence RNA quality. We summarize the comparative findings to guide researchers; detailed observations are provided in Table 6.

### 4.5. Limitations

Sample sizes per group were modest (n = 3), and the study did not include molecular assessments such as RNA or protein integrity measurements. Future studies may combine this anatomical workflow with downstream RNA, DNA, or protein quality-control metrics (such as RIN or DIN) using fresh tissue, and may extend anatomical validation to mouse models or to preparations intended for single-nucleus analyses in AHN research.

One limitation of the present study is that the dissections were performed by a single operator under standardized conditions. Although this approach ensured methodological consistency, microdissection procedures may be influenced by operator experience. Future studies involving multiple operators could help further assess reproducibility.

Another limitation is that the evaluation of the softening protocol was primarily based on macroscopic observations during the microdissection procedure, where anatomical boundaries of the dentate gyrus were identified through direct visual inspection. Although the protocol improved tissue pliability and facilitated the recognition of DG landmarks in archival fixed brain tissue, quantitative mechanical or image-based measurements (e.g., indentation resistance or grayscale-based contrast metrics) were not performed. Such quantitative indices could provide additional objective confirmation of the improvements in tissue handling and visibility. Future studies incorporating mechanical or image analysis-based metrics may further strengthen the evaluation of tissue softening protocols.

Although the present study was performed using rat brain tissue, many studies investigating adult hippocampal neurogenesis use mouse models. The general anatomical organization of the dentate gyrus is largely conserved across rodent species, including rats and mice, with a comparable laminar architecture composed of the molecular layer, granule cell layer, and hilus [30]. Nevertheless, as with all experimental animal systems, findings derived from rodent models should be interpreted with caution when considering their broader translational relevance [31].

## 5. Conclusions

We provide a validated, adoptable workflow for DG microdissection that (i) compares medial vs. coronal strategies under matched conditions, (ii) demonstrates comparable anatomical specificity across strategies and tissue states, and (iii) shows that a 15-day softening improves pliability and visibility of archival PFA-fixed tissue for training-oriented DG procurement. This workflow supports standardization of DG isolation, facilitates training without additional animal use in accordance with Reduction and Refinement principles, and strengthens the anatomical foundation required before conducting molecularly precise AHN studies on fresh tissue.

## Figures and Tables

**Table 1:** Experimental design showing brain hemisphere conditions (n = 3 per group), group codes, and subsequent experimental procedures.

Condition of the Brain Hemispheres	Group Codes	Sectioning Before DG Removal	Applied DG Removal Approach	Sectioning For Histological Evaluation After DG Removal
Fixed	Control	-	No DG Removal	+
Fixed	FXM	-	Medial	+
Fixed	FXC	+	Coronal	- *
Softened #	SM	-	Medial	+
Softened #	SC	+	Coronal	- *
Fresh	FM	-	Medial	+
Fresh	FC	+	Coronal	- *

“Fixed” refers to post-fixation in 4% paraformaldehyde (PFA) in phosphate-buffered saline (PBS) for 48 h following trans-cardiac perfusion with the same fixative. “Fresh” indicates immediate brain removal without fixation. FC fresh coronal, FM fresh medial, FXC fixed coronal, FXM fixed medial, SC softened coronal, SM softened medial. + Applied, - not applied. # Fixed brain hemispheres under slowly running tap water for 15 days. * Because the coronal slice had already been obtained prior to DG removal in these groups, histological evaluation was performed on the existing section without the need for additional slicing.

**Table 2:** Descriptive statistics for DG removal duration (s).

Approach	FM	FC	SM	SC
Total DG removal duration (s)	51.70 (6.51)	125.33 (8.62)	301.66 (12.34)	727.33 (16.62)

Total DG dissection durations in seconds among the groups. DG dentate gyrus, FC fresh coronal, FM fresh medial, SM softened medial, SC softened coronal.

**Table 3:** Independent *t*-test for DG removal duration.

Variable	T(df)	*p*	95% CI	η^2^p
FM vs. FC	−11.80 (4)	**0.00029**	[−90.95–56.30]	0.972
SM vs. SC	−35.61 (4)	**3.71 × 10^−6^**	[−458.85–392.47]	0.997
FM vs. SM	−31.01 (4)	**6.43 × 10^−6^**	[−272.34–227.59]	0.996
FC vs. SC	−55.68 (4)	**6.22 × 10^−7^**	[−632.01–571.98]	0.999

Bold font indicates a significant *p*-value of <0.05. CI confidence interval, DF degrees of freedom, FC fresh coronal, FM fresh medial, SM soften medial, SC softened coronal.

**Table 4:** Descriptive statistics of CA1–3 area sizes.

	Mean (SD)				
	Control	FM	FC	SM	SC
CA1	0.27 (0.023)	0.23 (0.02)	0.25 (0.049)	0.16 (0.02)	0.29 (0.03)
CA2	0.032 (0.002)	0.023 (0.002)	0.027 (0.008)	0.025 (0.01)	0.036 (0.002)
CA3	0.10 (0.014)	0.076 (0.008)	0.11 (0.026)	0.091 (0.037)	0.11 (0.017)

CA cornu ammonis, FC fresh coronal, FM fresh medial, SD standard deviation, SM softened medial, SC softened coronal.

**Table 5:** One-way ANOVAs.

Variable	F (DF, Error)	*p*	η^2^p
**CA1**	**2.36 (4, 10)**	**0.123**	**0.486**
**CA2**	**0.72 (4, 10)**	**0.597**	**0.224**
**CA3**	**0.642 (4, 10)**	**0.644**	**0.204**

F Indicates the statistic for ANOVA is the ratio of the mean square for the between groups divided by the mean square within groups. CA cornu ammonis, DF degrees of freedom.

**Table 6:** Comparative summary of medial and coronal dentate gyrus microdissection across fresh, fixed, and softened-fixed tissue states.

Tissue State	Approach	Dissection Duration (Relative)	Landmark Visibility	Tissue Pliability/Manipulation	Anatomical Specificity (Residual CA1–CA3)	Appropriate Use	Training Suitability Notes
Fresh	Medial	Faster (e.g., ~52 ± 6.5 s)	Clear anatomical borders	High pliability; single-block removal feasible	Comparable to coronal (ANOVA n.s.)	Minimize ex vivo time; rapid DG procurement	Requires familiarity with medial landmarks
Fresh	Coronal	Slower (e.g., ~125 ± 8.5 s)	Stepwise border identification; clear DV axis	Easy manipulation; controlled slicing	Comparable to medial (ANOVA n.s.)	Maximal border clarity; dorsal–ventral sampling	Longer procedure; requires slicing setup
Softened-fixed (15-day rinse)	Medial	Slower than fresh; faster than coronal (rank preserved)	Improved compared to fixed; less distinct than fresh	Improved pliability; some rigidity persists on medial surface	Comparable to coronal (ANOVA n.s.)	Archival tissue practice; anatomical training	Suitable for training only; not for molecular assays
Softened-fixed (15-day rinse)	Coronal	Slower than fresh	Markedly improved vs. fixed; borders well defined	Greatly increased pliability; controlled slicing	Comparable to medial (ANOVA n.s.)	Training; optimizing border definitions	Best visibility among fixed tissues; appropriate for stepwise teaching
Fixed (unsoftened)	Medial/ Coronal	Slowest	Low landmark visibility; reduced contrast	Rigid, brittle; difficult handling	Not evaluated for advantage	Not recommended;	Apply 15-day slow-running tap water protocol before use

Comparative summary of dentate gyrus (DG) microdissection performance across tissue states and dissection approaches. Dissection duration values reflect relative rankings; example mean ± SD values are provided for fresh tissue from the present dataset. For softened-fixed hemispheres, both approaches required longer durations than fresh tissue, although the medial < coronal ranking was preserved. Landmark visibility and tissue pliability indicate qualitative anatomical clarity based on direct observations. Anatomical specificity refers to residual CA1–CA3 areas after DG removal, which did not differ significantly between approaches (ANOVA, n.s.). Abbreviations: DG, dentate gyrus; CA, cornu ammonis; n.s., not significant (*p* > 0.05).

## Data Availability

The data (accessible part) presented in this study is available upon reasonable request to the corresponding author.

## Supplementary Materials

The following supporting information can be downloaded at: https://www.mdpi.com/article/10.3390/life16030511/s1, Supplementary Video S1: Fresh medial approach, Supplementary Video S2: Fixed medial approach, Supplementary Video S3: Softened medial approach, Supplementary Video S4: Fresh coronal approach, Supplementary Video S5: Fixed coronal approach, Supplementary Video S6: Softened coronal approach, Supplementary Video S7: Sectioning procedure.

## Abbreviations

The following abbreviations are used in this manuscript:

HP	Hippocampus
DG	Dentate gyrus
CA	Cornu ammonis
FC	Fresh coronal
FM	Fresh medial
SC	Softened coronal
SM	Softened medial
